# Prognostic significance of FOXP3+ tumor-infiltrating lymphocytes in breast cancer depends on estrogen receptor and human epidermal growth factor receptor-2 expression status and concurrent cytotoxic T-cell infiltration

**DOI:** 10.1186/s13058-014-0432-8

**Published:** 2014-09-06

**Authors:** Shuzhen Liu, William D Foulkes, Samuel Leung, Dongxia Gao, Sherman Lau, Zuzana Kos, Torsten O Nielsen

**Affiliations:** 1 Genetic Pathology Evaluation Centre, Department of Pathology and Laboratory Medicine, University of British Columbia, 509-2660 Oak Street, Vancouver, V6H 3Z6 BC Canada; 2 Program in Cancer Genetics, Department of Oncology and Human Genetics, McGill University, 3775 Chemin de la Côte-Sainte-Catherine, Montreal, H3T 1E2 QC Canada; 3 Department of Medical Genetics, Lady Davis Institute, Segal Cancer Center, Jewish General Hospital, 3775 Chemin de la Côte-Sainte-Catherine, Montreal, H3T 1E2 QC Canada; 4 Department of Medical Genetics, Research Institute of the McGill University Health Center, 1650 Cedar Avenue, Montreal, H3G 1A4 QC Canada; 5 Department of Pathology and Laboratory Medicine, University of Ottawa, 451 Smyth Road, Ottawa, K1H 8 M5 ON Canada; 6 Department of Laboratory Medicine and Pathobiology, University of Toronto, 27 King’s College Circle, Toronto, M5S 1A1 ON Canada

## Abstract

**Introduction:**

The infiltration of FOXP3^+^ regulatory T cells into invasive tumors has been reported to be associated with survival in a variety of cancers. The prognostic significance of FOXP3^+^ tumor-infiltrating lymphocytes (TILs) in breast cancer, however, remains controversial.

**Methods:**

FOXP3^+^ TILs were assessed by immunohistochemistry on tissue microarrays constructed from a well-defined cohort of 3,992 breast cancer patients linked to detailed demographic, biomarker, treatment and outcome data. Survival analyses were performed using the Kaplan-Meier function and Cox proportional hazards regression models to evaluate the association of FOXP3^+^ TILs with breast cancer-specific survival, stratified by intrinsic subtype and cytotoxic T-cell infiltration status (as defined by CD8 immunohistochemistry).

**Results:**

The presence of high numbers of FOXP3^+^ TILs was significantly associated with young age, high grade, estrogen receptor (ER) negativity, concurrent CD8^+^ cytotoxic T-cell infiltration, and human epidermal growth factor receptor-2 positive (HER2^+^)/ER^+^ and core basal subtypes. On multivariate survival analysis, a high level of FOXP3^+^ TILs was significantly associated with poor survival in ER^+^ breast cancers that lacked CD8^+^ T-cell infiltrates (hazard ratio (HR) = 1.30, 95% confidence interval (CI) = 1.02 to 1.66). However, in ER^+^ breast cancers, FOXP3^+^ TILs were strongly associated with improved survival in the HER2^+^/ER^+^ subgroup, particularly in those with co-existent CD8^+^ T-cell infiltrates (HR = 0.48, 95% CI = 0.23 to 0.98), for which the presence of high levels of FOXP3^+^ TILs was independent of standard clinical prognostic factors.

**Conclusions:**

FOXP3^+^ regulatory TILs are a poor prognostic indicator in ER^+^ breast cancer, but a favorable prognostic factor in the HER2^+^/ER^+^ subtype. The prognostic value of FOXP3^+^ TILs in breast cancer differs depending on ER and HER2 expression status and CD8^+^ T-cell infiltration.

**Electronic supplementary material:**

The online version of this article (doi:10.1186/s13058-014-0432-8) contains supplementary material, which is available to authorized users.

## Introduction

FOXP3 is a forkhead box transcription factor, containing a DNA-binding domain that can recruit both transcriptional activator and repressor complexes to target genes [1]. This transcription factor plays an important role in the development and function of immune regulatory T cells (Tregs), and can be used as a specific biomarker for the identification of Tregs within an inflammatory infiltrate [2]. Tregs are critical for the maintenance of self-tolerance. There is also mounting evidence that these cells play a central role in immune tolerance to tumor cells by several mechanisms, including inhibiting effector cytotoxic T-cell lymphocytes by reversibly interfering with the release of lytic granules by CD8+ T cells, thereby impeding target cell lysis [3]. Effective evasion of the immune system by tumor cells is necessary during oncogenesis, tumor progression and metastatic spread. Increased activity of Tregs has been linked with a poor immunological response to tumor antigens and is thought to represent a critical mechanism of immune evasion by tumors. The tumor microenvironment has been reported to contain a rich milieu of molecules capable of increasing the number of FOXP3+ Tregs by several possible mechanisms, including driving CD4+ T-helper cells to develop into FOXP3+ Tregs, recruiting existing FOXP3+ Tregs to the tumor site, and inducing the expansion of resident Tregs. This tumor-induced increase in FOXP3+ Tregs represents a potential barrier to attempts at cancer immunotherapy [4],[5].

Studies addressing the prognostic significance of FOXP3+ Tregs have shown conflicting results. The presence of FOXP3+ tumor-infiltrating lymphocytes (TILs) has been reported to be associated with poor clinical outcome in a variety of cancer types, including prostatic, lung, hepatocellular and renal cell carcinomas [6]-[10], indicating that cancer patients may benefit from blocking the capacity of tumor cells to recruit Tregs. Conversely, other studies have found that FOXP3+ TILs correlate with favorable prognosis in colorectal, gastric, ovarian and head and neck carcinomas [11]-[15]. These discrepant prognostic associations of FOXP3+ TILs reflect the complexity of biological processes affecting the host immunological response to tumoral tissue – in some tumors, immune infiltrates are recruited by tumor cells and facilitate tumor spread, whereas in other tumors immune infiltrates reflect a host anti-tumor reaction. The type of T cell present may help distinguish between these types of responses, but this requires subtyping of TILs into regulatory (FOXP3+) and cytotoxic (CD8+) populations. For example, we recently showed that the presence of CD8+ cytotoxic T-cell infiltrates in breast cancer is a good prognostic factor in basal breast cancers, but not in the other intrinsic molecular subtypes of breast cancer [16].

Even within breast cancer, the prognostic significance of FOXP3+ TILs has been widely debated. Recent studies have reported that FOXP3+ T-cell infiltration is associated with poor clinical outcome [17]-[22], whereas others found no significant prognostic role for FOXP3+ infiltration in a large series of breast cancers [23]. Indeed, some recent evidence suggests that FOXP3+ TILs could actually be a favorable survival indicator in certain subgroups. A recent study, using a cohort of 175 estrogen receptor (ER)-negative breast cancers found that FOXP3+ TILs were positively correlated with the concurrent presence of CD8+ TILs, and were an independent good prognostic factor in ER-negative breast cancer [24]. Another recent study reported that FOXP3+ TILs were a significant and independent factor associated with improved overall survival and progression-free survival in triple-negative breast cancer [25]. The properties of FOXP3+ TILs may be affected by the tumor microenvironment, thus the prognostic value of FOXP3+ TILs could possibly be influenced by molecular subtype and interactions with other immune cells. To date, few studies have had sufficient power to investigate the prognostic effect of the interaction of FOXP3+ TILs with molecular subtypes or different types of immune response in breast cancer.

We implemented this study to evaluate the prognostic significance of FOXP3+ TILs, in a population-based breast cancer cohort with long-term follow-up and detailed biomarker data defining the main intrinsic molecular subtypes and cytotoxic T-cell infiltrates in breast tumor tissues.

## Materials and methods

### Study cohort

The study population has been previously described [16],[26],[27]. In brief, the primary cohort in this study includes 3,992 female patients diagnosed with invasive breast cancer between 1986 and 1992 in the province of British Columbia, representing roughly 20% of all patients diagnosed with breast cancer in the province during that time period. The mean age of the population at diagnosis of breast cancer was 58.9 years (range, 23 to 95 years). The median follow-up length was 12.6 years (range, 0.1 to 18.5 years). Basic clinical and pathological characteristics of the study cohort are summarized in Table 1. This study and our access to the de-identified data were approved by the Clinical Research Ethics Board of the University of British Columbia and British Columbia Cancer Agency. No informed consent was needed as anonymized archival specimens were used in this study, according to the Canadian Tri-Council Policy Statement for ethical research involving human subjects.

**Table 1:** **Clinicopathologic characteristics and distribution of FOXP3+ intratumoral lymphocytes (iTILs) in the study population**

Characteristics	Number of patients	FOXP3+ iTILs (≥2)	
	(%)	Prevalence %	***P***value
**Age at diagnosis (year)**			<0.001
<40	294 (7.4)	41.6 (106/255)	
40-49	844 (21.1)	38.5 (264/685)	
50-65	1,425 (35.7)	29.5 (350/1186)	
>65	1,429 (35.8)	27.0 (311/1150)	
**Grade**			<0.001
1 (well differentiated)	209 (5.2)	16.3 (25/153)	
2 (moderately well or partially differentiated)	1,563 (39.2)	23.7 (305/1288)	
3 (poorly differentiated)	2,040 (51.1)	38.9 (661/1699)	
Unknown	180 (4.5)		
**Tumor size (cm)**			0.201
≤2	2,078 (52.1)	30.2 (512/1697)	
>2-5	1,667 (41.8)	33.1 (461/1394)	
> 5	221 (5.5)	29.8 (50/168)	
Unknown	26 (0.6)		
**Nodal status**			0.003
Negative	2,265 (56.7)	29.4 (545/1856)	
Positive	1,719 (43.1)	34.2 (484/1414)	
Unknown	8 (0.2)		
**Lymphovascular invasion (LVI)**			0.218
Negative	2,106 (52.8)	30.3 (522/1720)	
Positive	1,710 (42.8)	32.4 (461/1423)	
Unknown	176 (4.4)		
**AJCC stage**			0.003
I	1,393 (34.9)	27.7 (314/1132)	
II	2,255 (56.5)	33.7 (630/1872)	
III	317 (7.9)	32.2 (82/255)	
Unknown/missing	27 (0.7)		
Adjuvant systemic therapy (AST)*			<0.001
No AST	1,676 (42.0)	29.0 (402/1386)	
Tamoxifen only	1,276 (32.0)	27.7 (287/1037)	
Chemotherapy only	727 (18.2)	40.6 (245/603)	
Tamoxifen + chemotherapy	297 (7.4)	38.8 (92/237)	
Other	16 (0.4)	35.7 (5/14)	
**ER**			<0.001
Negative	1,200 (30.1)	42.7 (388/909)	
Positive (≥1% nuclei stained)	2,761 (69.1)	27.2 (641/2354)	
Uninterpretable/missing	31 (0.8)		
**HER2**			<0.001
Negative	3,316 (83.1)	29.9 (830/2779)	
Positive	498 (12.5)	42.4 (184/434)	
Uninterpretable/missing	178 (4.4)		
**Subtype**			<0.001
Luminal A	1,518 (38.0)	20.8 (275/1325)	
Luminal B	829 (20.8)	36.8 (276/749)	
Luminal/HER2	224 (5.6)	39.5 (79/200)	
Luminal not further assigned	244 (6.1)	19.5 (29/149)	
HER2+/ER–	250 (6.3)	45.9 (102/222)	
TNP	630 (15.8)	42.2 (226/535)	
Core basal	330 (8.3)	54.0 (163/302)	
5NP	162 (4.1)	36.5 (50/137)	
TNP not assignable	138 (3.4)	39.5 (34/86)	
Unassignable	297 (7.4)	21.5 (23/107)	
**CD8+ iTIL**			<0.001
0	2,299 (57.6)	19.6 (410/2094)	
≥1	1,104 (27.6)	56.6 (591/1044)	
Uninterpretable/missing	589 (14.8)		
**Total**	**3,992 (100)**	**31.5 (1031/3277)**	

*Chemotherapy was methotrexate, cyclophosphamide, 5-fluorouracil (CMF), doxorubicin and cyclophosphamide (AC) or doxorubicin, cyclophosphamide, 5-fluorouracil (FAC). Trastuzumab was not available to these patients. 5NP, five-marker negative phenotype; AJCC, American Joint Committee on Cancer; ER, estrogen receptor; HER2, human epidermal growth factor receptor-2; iTILs, intratumoral tumor-infiltrating lymphocytes; TNP, triple-negative phenotype.

### Immunohistochemistry

Archival formalin-fixed paraffin-embedded tissue blocks for each of the 3,992 patients were retained in the centralized provincial laboratory at the Vancouver General Hospital, which performed ER evaluations during that time period. Hematoxylin and eosin-stained slides from these blocks were reviewed by two pathologists to identify the invasive breast carcinoma area, and the representative areas containing the most densely cellular viable invasive breast carcinoma tissue were chosen for coring. Tissue microarrays (TMAs) were constructed from these samples and were assessed by immunohistochemical (IHC) staining for ER, progesterone receptor (PR), human epidermal growth factor receptor-2 (HER2), Ki67, epidermal growth factor receptor (EGFR), cytokeratins 5/6 (CK5/6) and CD8 as previously described [16],[26],[28]-[32]. Breast cancer subtypes were defined by the IHC expression profile of ER, PR, HER2 (reported as per the 2007 American Society of Clinical Oncology/College of American Pathologists (ASCO/CAP) guidelines) [33], Ki67, EGFR and CK5/6 as previously described [16],[29] and were categorized in Table 1. Assessment of CD8+ TILs has been previously reported [16]. IHC staining for FOXP3+ TILs was performed using the anti-human FOXP3 monoclonal mouse antibody (clone 236A/E7, (Abcam, Cambridge, MA, USA) dilution 1:20) according to the manufacturer’s instruction. The expected nuclear staining pattern was optimized on benign lymph node tissue, which was also used as the positive control for each batch and to assess technical consistency of staining between batches. Images of the FOXP3-stained TMA cores are available at our TMA image website [34].

### FOXP3+ TIL measurement

Scoring of FOXP3+ TILs was performed using digitally scanned TMA slides by a single pathologist blinded to the results of other markers, clinical characteristics and outcomes of the patients. FOXP3+ `intratumoral’ tumor-infiltrating lymphocytes (iTILs) were defined as FOXP3-stained lymphocytes present within the malignant epithelial cell nests, while FOXP3+ `stromal’ tumor-infiltrating lymphocytes (sTILs) were defined as FOXP3-stained lymphocytes in the peritumoral stroma, lacking direct contact with the epithelial cancer cells. The counts of combined iTILs and sTILs were recorded as `total’ FOXP3+ tumor-infiltrating lymphocytes (tTILs) [16],[35]. To test the reproducibility of our scoring system, 281 cases were randomly selected from the whole cohort, and FOXP3+ iTILs and sTILs were rescored by the same pathologist, as well as by a second pathologist blinded to the original scores. We observed high reproducibility of FOXP3+ TILs scoring by the same scorer (Pearson correlation coefficient = 0.91 for FOXP3+ iTILs and 0.97 for FOXP3+ sTILs), and between the two scorers (intraclass correlation coefficient = 0.88 for FOXP3+ iTILs and 0.90 for FOXP3+ sTILs).

### Statistical analysis

Statistical analyses were performed using SPSS version 21.0 (IBM Corp, Armonk, NY, USA) and R version 2.11.1 [36]. The prespecified primary endpoint of this study was breast cancer-specific survival (BCSS), defined as the time from the date of initial diagnosis of breast cancer to the date of death attributed to breast cancer. Death from another cause or alive at end of follow-up were treated as censored events. We also performed supplementary analyses using relapse-free survival (RFS) as the outcome. RFS time was defined as the number of years from the date of diagnosis of breast cancer to the date of any type of relapse of the disease. Survival curves were generated using the Kaplan-Meier method, and BCSS differences among subgroups were evaluated for significance using the log-rank test. Cox proportional hazards regression models were built for multivariate survival analyses to estimate the hazard ratio (HR) of FOXP3+ TILs adjusted by potential confounding factors, including age at diagnosis, tumor grade, tumor size, lymph node status, lymphovascular invasion, and intrinsic subtype. The Wald test was used to evaluate the significance of individual coefficients in the models. Proportional hazard assumptions were examined by smoothed rescaled residuals plots. Interaction between FOXP3+ and CD8+ TILs, with respect to the association with survival outcome, was tested by building Cox regression models at different status of CD8+ iTILs, and then evaluating the hazard ratios of FOXP3+ TILs. The prespecified cutoff point for CD8+ iTIL (0 vs. ≥1) used for the analyses was validated in our previous studies [16],[35]. All tests were two-sided with a significance level at α = 0.05.

Repeated random subsampling training test was used to identify and validate an optimal cutoff point for FOXP3+ TILs by means of receiver operating characteristic (ROC) curve analysis [37] and Youden Index, using the 10-year BCSS as the endpoint, as described in our previous study [16]. Briefly, ROC curves were used to show the sensitivity and specificity for the 10-year BCSS for each value of FOXP3+ TILs imputed into the equation. The training set was randomly selected to include 50% of the total study population and was used to identify an optimal cutoff point by ROC analysis, which was then tested on the remaining 50% (test set) of the study population. The Youden Index was applied to determine if the selected cutoff was valid in the test set. This procedure was repeated 100 times to ensure robustness (Table S1 in Additional file 1). Since BCSS is a time to event endpoint, we also used X-tile software (version 3.6.1, Yale University School of Medicine, New Haven, CT, USA) to validate the selected cutoff points. The optimal cutoffs for FOXP3+ TILs identified in these analyses were 2 for iTILs, 3 for sTILs and 4 for tTILs. In this study, FOXP3+ iTILs were categorized as low when <2 FOXP3+ iTILs were identified in a single tissue core, and as high when ≥2 FOXP3+ iTILs were identified. FOXP3+ sTILs were categorized as low when <3 FOXP3+ sTIL were identified in a single tissue core and as high when ≥3 FOXP3+ sTILs were identified. Lastly, FOXP3+ tTILs were categorized as low when <4 FOXP3+ tTIL were identified in a single tissue core and as high when ≥4 FOXP3+ tTILs were identified.

## Results

### Distributions and prognosis of FOXP3+ TILs in the whole study cohort

Of the 3,992 breast cancer patients in the original study cohort, 3,277 (82.1%) cases had intact 0.6 mm TMA cores containing infiltrating breast carcinoma and were interpretable for FOXP3 staining. At least one FOXP3+ iTIL (intratumoral) was present in 45.0% and at least one sTIL (stromal) in 74.8% of the 3,277 tumor cases. Both FOXP3+ iTILs and sTILs were present in the same core in 42% of cases (sample images shown in Figure S1 in Additional file 2). The median number of FOXP3+ TILs was 0 for iTILs (interquartile range IQR, 0 to 2), 3 for sTILs (IQR, 3 to 11), and 4 for tTILs (IQR, 4 to 13). In this paper, we report the results from the FOXP3+ iTIL primary analysis as this measurement is fastest and simple to perform, and was the primary analysis previously applied to CD8+ TILs on this population; results from stromal and total categorizations of TILs were generally similar. Tumors with ≥2 FOXP3+ iTIL were significantly correlated with young age, high grade, positive nodal status, the presence of CD8+ iTILs, ER negativity, and with the HER2+/ER − and the core basal intrinsic subgroups (Table 1).

**Figure 1:**
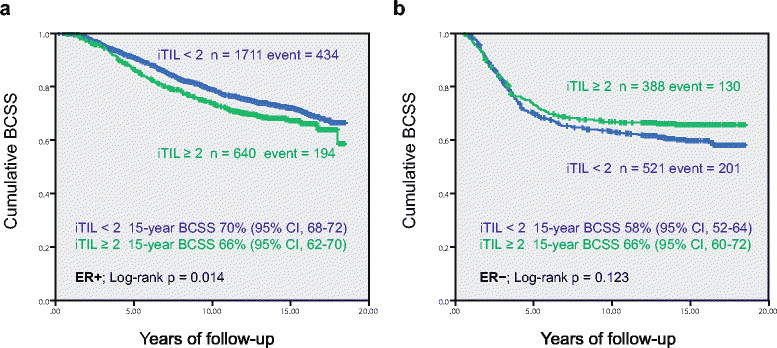
**Breast cancer-specific survival (BCSS) stratified by FOXP3+ iTILs in ER-positive and ER-negative breast cancers. (a)** ER+, **(b)** ER–. ER, estrogen receptor; iTILs, intratumoral tumor-infiltrating lymphocytes.

To assess the prognostic value of FOXP3+ iTILs in the whole study cohort, we built Cox proportional hazards regression models to estimate the hazard ratio of FOXP3+ iTIL for breast cancer specific survival. Neither the univariate nor multivariate Cox regression analyses showed a significant association of FOXP3+ iTIL with BCSS in the whole cohort of unselected breast cancer patients (univariate analysis: HR = 1.13, 95% confidence interval (CI) = 0.99 to 1.29; multivariate analysis: HR = 0.89, 95% CI = 0.77 to 1.02; full details in Table S2 in Additional file 1). Kaplan-Meier survival analysis revealed that the prognostic significance of FOXP3+ iTILs was subtype-specific and was associated with opposite prognostic effects in ER positive versus ER negative tumors. As shown in Figure 1, FOXP3+ iTILs were an indicator of poor survival in ER − tumors, but a favorable prognostic indicator in ER − breast cancers, indicating that the interaction of FOXP3+ iTILs with ER status could mitigate the prognostic effect of FOXP3+ TILs in the unstratified breast cancer population.

### Prognosis of FOXP3+ iTILs in ER positive breast cancer

Although the Kaplan-Meier curves (Figure 1) showed that a high level of FOXP3+ iTILs (≥2) was significantly associated with worse BCSS in ER + breast cancer (*P* = 0.015), on multivariate Cox regression analysis the hazard ratio for FOXP3+ iTILs was not independently significant in this group (HR = 1.14, 95% CI = 0.96 to 1.37; *P* = 0.14), when adjusted for age, tumor grade, tumor size, nodal status, and lymphovascular invasion (Table 2). We then evaluated the prognostic effect of the interaction of FOXP3+ iTILs with CD8+ cytotoxic T-cell infiltrates in ER + breast cancers by building multivariate Cox regression models stratified by CD8+ iTIL status. The stratified multivariate regression analyses showed that the poor prognostic effect of FOXP3+ iTILs in the ER + group was only detected in those with CD8+ iTIL = 0 (HR = 1.30, 95% CI = 1.02 to 1.66; *P* = 0.03, Table 2).

**Table 2:** **Estimates of hazard ratio with multivariate analysis in ER + breast cancers, with different CD8+ iTIL status**

Variable	ER + (n = 2166)		ER+/CD8+ iTIL = 0 (n = 1474)		ER+/CD8+ iTIL ≥1 (n = 624)	
	HR (95% CI)	*P*	HR (95% CI)	*P*	HR (95% CI)	*P*
**Age**	1.06	0.536	1.03	0.803	1.14	0.449
≥50 vs. <50	(0.88 - 1.27)		(0.82 - 1.29)		(0.82 - 1.59)	
**Grade**	1.67	<0.001	1.67	<0.001	1.71	0.001
3 vs. (1 and 2)	(1.41 - 1.99)		(1.35 - 2.05)		(1.24 - 2.36)	
**Tumor size**	1.76	<0.001	1.75	<0.001	1.66	0.002
>2 cm vs. ≤2 cm	(1.48 - 2.10)		(1.41 - 2.16)		(1.21 - 2.27)	
**Nodal status**	1.98	<0.001	2.18	<0.001	1.51	0.017
Positive vs. negative	(1.63 - 2.40)		(1.72 - 2.77)		(1.08 - 2.13)	
**LVI**	1.30	0.007	1.33	0.020	1.24	0.209
Positive vs. negative	(1.07 - 1.57)		(1.05 - 1.68)		(0.89 - 1.74)	
**FOXP3+ iTIL**	1.14	0.138	1.30	0.032	1.00	0.978
≥2 vs. <2	(0.96 - 1.37)		(1.02 - 1.66)		(0.74 - 1.34)	

CI, confidence interval; ER, estrogen receptor; HR, hazard ratio; iTIL, intratumoral tumor-infiltrating lymphocyte; LVI, lymphovascular invasion.

### Prognosis of FOXP3+ iTILs in ER negative breast cancer

**Figure 2:**
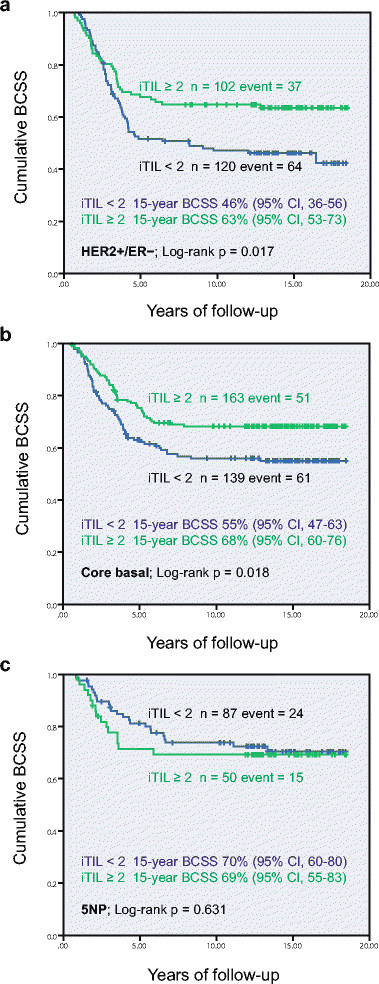
**Breast cancer-specific survival (BCSS) stratified by FOXP3+ iTILs in different subtypes of ER– breast cancer. (a)** HER2+/ER–, **(b)** core basal, and **(c)** five-marker negative phenotype (5NP) subgroup. ER, estrogen receptor; HER2, human epidermal growth factor receptor-2; iTILs, intratumoral tumor-infiltrating lymphocytes.

Because standard six-marker IHC analysis, as used in this study, can subdivide ER– breast cancers into the distinct subtypes of HER2+/ER–, core basal and five-marker negative phenotype (5NP), each with distinct underlying biology, we assessed the prognosis of FOXP3+ iTILs in these subtypes separately in a preplanned stratification. The Kaplan-Meier analysis showed that, in the HER2+/ER– and core basal intrinsic subtypes, the differences in BCSS were significant between patients with high and low FOXP3+ iTILs (Figure 2a and b). Cases in these two subtypes with high levels of regulatory T cells (that is FOXP3+ iTIL ≥2) had significantly better survival than those with FOXP3+ iTIL <2. However, in the 5NP subtype, constituting cases labeled as `triple negative’ but not expressing positive markers of the basal group, no such association or even trend was observed (Figure 2c).

**Figure 3:**
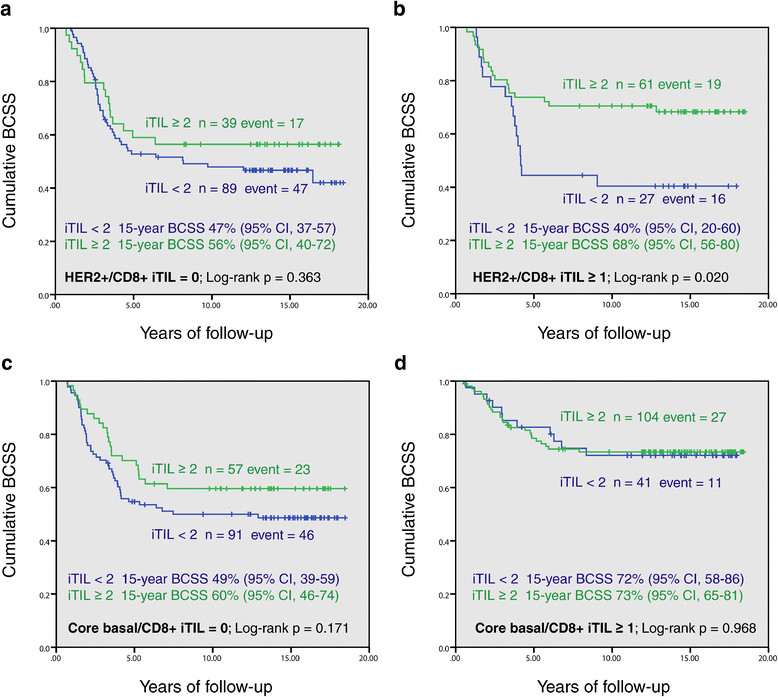
**Breast cancer-specific survival (BCSS) by FOXP3+ iTILs in HER2+/ER– and core basal intrinsic subtypes, stratified by CD8+ iTILs status. (a)** HER2+/ER– with no CD8+ iTILs, **(b)** HER2+/ER– with presence of CD8+ iTILs, **(c)** core basal with no CD8+ iTILs, and **(d)** core basal with presence of CD8+ iTILs. ER, estrogen receptor; HER2, human epidermal growth factor receptor-2; iTILs, intratumoral tumor-infiltrating lymphocytes.

We assessed the prognostic effect of the interaction between FOXP3+ (regulatory) and CD8+ (cytotoxic) iTILs in the HER2+/ER– and core basal subtypes, by performing Kaplan-Meier survival analyses incorporating the CD8+ iTILs status. It was demonstrated that the improved prognosis associated with FOXP3+ iTIL was only significant in the HER2+/ER– subtype when CD8+ iTILs were concurrently present. Among these cases, at 15 years of follow-up, patients with FOXP3+ iTIL ≥2 had a 28% higher cumulative survival rate than those with FOXP3+ iTIL <2 (Figure 3b). No significant associations were observed in other subgroups (Figure 3a, c and d), despite larger numbers of patients.

We further built multivariate Cox regression models including the conventional clinical co-variables to assess the independent prognostic value of the regulatory FOXP3+ iTILs in the major subtypes of ER-negative breast cancers, stratified by the presence or absence of concurrent cytotoxic CD8+ iTILs. Table 3 showed that in the HER2+/ER– subtype, when CD8+ iTILs are present, patients with FOXP3+ iTIL ≥2 had a significantly (52%, 1 to 0.48) higher probability of survival than those with FOXP3+ iTIL <2. Although the favorable effect was also observed in those lacking this evidence of concurrent CD8+ T-cell infiltration, the magnitude of difference was smaller and not significant (HR = 0.84, 95% CI = 0.48 to 1.46, *P* = 0.36). However, in the core basal subtype, although we observed a significant favorable prognostic effect of FOXP3+ iTILs in general, after taking CD8+ iTIL into account, the effect became insignificant. More interestingly, both the univariate Kaplan-Meier function and multivariate Cox regression analyses showed that FOXP3+ iTILs did not have apparent prognostic effect in the core basal cases with CD8+ T-cell infiltrates (HR = 0.93, 95% CI = 0.43 to 2.00; *P* = 0.85). The favorable prognostic effect of FOXP3+ iTILs observed in the whole core basal subtype could be primarily attributable to the concurrent high presence of CD8+ iTILs, as observed in our previous study [16].

**Table 3:** **Estimates of hazard ratio with multivariate analysis in HER2+/ER– and core basal subtypes, stratified by CD8+ iTIL status**

Variable	Whole subgroup		With CD8+ iTIL = 0		With CD8+ iTIL ≥ 1	
	HR (95% CI)	*P*	HR (95% CI)	*P*	HR (95% CI)	*P*
***HER2+/ER–***						
**Age**	1.13	0.573	1.09	0.741	1.12	0.784
≥50 vs. <50	(0.74 - 1.73)		(0.65 - 1.84)		(0.50 - 2.50)	
**Grade**	2.23	0.007	2.05	0.039	2.55	0.134
3 vs. (1 and 2)	(1.24 - 4.01)		(1.04 - 4.07)		(0.75 - 8.65)	
**Tumor size**	1.67	0.026	1.43	0.198	2.53	0.025
>2 cm vs. ≤2 cm	(1.06 - 2.61)		(1.83 - 2.48)		(1.13 - 5.67)	
**Nodal status**	1.95	0.008	1.20	0.588	3.87	0.002
Positive vs. negative	(1.19 - 3.21)		(0.62 - 2.35)		(1.67 - 8.96)	
**LVI**	1.34	0.250	1.47	0.261	1.56	0.268
Positive vs. negative	(0.82 - 2.19)		(0.75 - 2.85)		(0.71 - 3.40)	
**FOXP3+ iTIL**	0.71	0.104	0.84	0.528	0.48	0.047
≥2 vs. <2	(0.46 - 1.07)		(0.48 - 1.49)		(0.23 - 0.98)	
***Core basal***						
**Age**	1.01	0.957	0.77	0.301	1.53	0.245
≥50 vs. <50	(0.68 - 1.50)		(0.47 - 1.26)		(0.75 - 3.12)	
**Grade**	1.30	0.417	1.55	0.257	1.91	0.387
3 vs. (1 and 2)	(0.69 - 2.44)		(0.73 - 3.28)		(0.44 - 8.22)	
**Tumor size**	1.62	0.020	2.81	<0.001	0.77	0.470
>2 cm vs. ≤2 cm	(1.08 - 2.43)		(1.67 - 4.72)		(0.38 - 1.57)	
**Nodal status**	2.37	<0.001	2.95	<0.001	1.64	0.212
Positive vs. negative	(1.53 - 3.66)		(1.68 - 5.17)		(0.75 - 3.56)	
**LVI**	1.37	0.159	0.86	0.604	3.60	0.003
Positive vs. negative	(0.88 - 2.13)		(0.49 - 1.51)		(1.56 - 8.29)	
**FOXP3+ iTIL**	0.53	0.002	0.66	0.130	0.93	0.845
≥2 vs. <2	(0.36 - 0.79)		(0.39 - 1.13)		(0.43 - 2.00)	

CI, confidence interval; ER, estrogen receptor; HR, hazard ratio; HER2, human epidermal growth factor receptor-2; iTIL, intratumoral tumor-infiltrating lymphocyte; LVI, lymphovascular invasion.

## Discussion

The infiltration of tumors by regulatory T cells (defined as FOXP3+ TILs) has been reported to be associated with patient survival in a variety of cancers, but its prognostic value remains controversial. We evaluated the prognostic effect of FOXP3+ TILs in breast cancer, using a large population-based breast cancer cohort with long clinical follow-up. In addition to assessing the independent prognostic value of FOXP3+ TILs, adjusted by conventional clinicopathological confounding factors, we also investigated the prognostic effect of the interaction between regulatory and cytotoxic T-cell infiltrates (defined as CD8+ TILs) in different molecular subtypes of breast cancer, a novel analysis made possible by the large sample size and availability of concurrent biomarker data. Results from this study demonstrate that, in breast cancer, the presence of high levels of FOXP3+ TILs is associated with young age, high grade, positive nodal status, concurrent CD8+ T-cell infiltrates, and ER negativity (including both the HER2+/ER– and core-basal subtypes). In ER + breast cancer, high FOXP3+ TILs were significantly associated with poor clinical outcome. When ER + tumors did not display concurrent CD8+ lymphocyte infiltration, patients with high levels of FOXP3+ TILs had 30% higher cumulative death rates than those with low FOXP3+ TILs. However, in ER– group, the prognostic effect of FOXP3+ TILs differed by intrinsic subgroup, depending on the presence of CD8+ T-cell infiltration. In HER2+/ER– breast cancer, the multivariate analysis indicates that high FOXP3+ lymphocyte infiltration is a significant, independent and favorable prognostic factor in patients having tumors with CD8+ T-cell infiltrates. In this subgroup, those with high levels of FOXP3+ TILs had 52% higher probability of survival than those with low FOXP3+ TILs. However, in the core-basal subgroup, although high levels of FOXP3+ lymphocyte infiltration was associated with improved survival in general, the presence of CD8+ T-cell infiltrates appeared to be the stronger and more important favorable prognostic factor.

The prognostic associations of FOXP3+ TILs could be affected by tumor microenvironment, including tumor site, histologic and molecular subtype, and different types of immune response, all of which may interactively influence clinical outcome of cancer patients. In general, patients with ER + breast cancers experience better clinical outcome than those with ER– tumors, but not all ER + breast cancer patients have good survival. It was reported that FOXP3+ TILs were associated with poor clinical outcome in ER + breast cancer [17],[23]. The multivariate analysis from our study confirms that, in ER + breast cancer, FOXP3+ regulatory TILs are an indicator for poor survival, at least in those tumors lacking concurrent cytotoxic CD8+ T-cell infiltration. One possible explanation consistent with these findings is that FOXP3+ TILs reflect tumor-induced immune evasion in ER + breast cancers. Although patients with ER– tumors usually have poor survival, some biological signatures serve as favorable prognostic indicators among ER– breast cancers. Studies with clinical and gene expression data demonstrate that an immune response is significantly associated with improved prognosis among ER– breast cancers [38]-[41]. In our study, FOXP3+ TILs were found to be a favorable indicator of survival in ER– breast cancer, which is also consistent with the findings from other recently published studies [24],[25]. Cause and effect cannot be determined from this type of association study, but for the HER2+/ER– group at least one interpretation consistent with the data is that FOXP3+ TILs are induced secondarily by a robust antitumor CD8+ TIL response.

Until now, comparatively few studies have been powered to assess if different categories of tumor-infiltrating lymphocytes influence clinical outcomes in different breast cancer molecular subtypes. Our study indicates that the infiltration of FOXP3+ lymphocytes not only has distinctive prognostic associations within different subtypes of breast cancer, but also interacts with cytotoxic T-cell infiltrates in their association with patient survival. Our multivariate analyses stratified for presence or absence of CD8+ T-cell infiltration demonstrated that the favorable prognostic effect of FOXP3+ TILs in ER– breast cancer was only significant and independent in those having HER2+/ER– tumors with concurrent CD8+ T-cell infiltrates. In the core basal subtype, the favorable prognostic effect associated with a TIL immune response may be primarily due to CD8+ T-cell infiltration, which may be counteracted when FOXP3+ Tregs are also present. Despite our large sample size and event rate with long follow-up, these subgroup analyses still have limited power, and as a retrospective cohort analysis will require external validation or incorporation into meta-analyses to increase the level of evidence for these results.

The prognostic significance of immune responses in breast cancer is controversial. Most of the studies reporting that FOXP3+ TILs are an indicator of poor prognosis in breast cancer applied unstratified survival analyses [17],[19] and might be expected to largely reflect the majority ER + population, possibly confounded by an opposite association in ER– cases. Recent studies, using large breast cancer cohorts, have consistently demonstrated that cytotoxic T-cell infiltration plays different prognostic roles in different molecular subtypes [16],[35],[42]. To our knowledge, ours is the first study with sufficient power to evaluate the prognosis of these interactions stratified by subtype and cytotoxic vs. regulatory lymphocyte infiltration. Some studies have applied ratios of CD8+: FOXP3+ TILs to evaluate the prognostic effect of immune response in breast cancer, and reported that those with a ratio of CD8+: FOXP3+ TILs ≥1 had better survival than those with ratios <1 [21],[24],[43]. The results from our study suggest that both FOXP3+ and CD8+ T-cell infiltration have distinctive prognostic implications in different molecular subtypes of breast cancer. A simple ratio between FOXP3+ and CD8+ TILs may not be an appropriate universal prognostic indicator, across biologically different breast cancer subtypes.

Inconsistency in defining and measuring tumor infiltrating lymphocytes may also underlie apparently discrepant research results. In this study, we used specific immunohistochemistry with a widely used mouse monoclonal anti-human FOXP3 antibody to detect regulatory intratumoral and stromal TILs in breast tumor tissue. Both ROC curve and X-tile methods were applied to define the optimal cutoff points of FOXP3+ TILs for the survival analyses. These findings apply to the TMA platform but would need modification and re-validation to be applied to whole sections. To confirm the prognosis of FOXP3+ TILs in breast cancer, we also evaluated the association of FOXP3+ iTIL with relapse-free survival; results were very similar to those using breast cancer-specific survival as the outcome (Tables S3-S5 in Additional file 1). Furthermore, we tested the correlation of FOXP3+ sTILs and tTILs with patient clinical and pathological characteristics. As with the primary assessment of intratumoral lymphocytes, results demonstrated positive correlations between presence of high numbers of FOXP3+ sTIL and tTIL with young age, high grade, positive nodal status, CD8+ T-cell tumor infiltration, ER negativity, and with HER2+/ER– and core basal subgroups (Table S6 in Additional file 1).

Although we believe that the measurement of FOXP3+ TILs and the evaluation of the association of FOXP3+ TILs with breast cancer patient survival are reliable and form a consistent picture with other reports, there are some limitations in this study. Studies suggest that FOXP3 represents the most specific marker for Treg cells, but some rare subtypes of cytotoxic lymphocytes may also express FOXP3 [17],[44]. This study only assessed the interaction between FOXP3+ and CD8+ T-cell infiltration in breast cancer; further studies need to be done to differentiate other types of chronic inflammatory infiltration. Studies have reported that pre-existing immune responses may fortify the effect of chemotherapy [45],[46]. TILs could be a favorable predictive indicator for targeted trastuzumab therapy [47],[48]. The patients in our study predate the trastuzumab era, and the types of endocrine and chemotherapy given would be considered outdated by contemporary standards of care. An exploratory multivariate analysis (Table S7 in Additional file 1) found that pre-existing FOXP3+ Treg infiltration might be a favorable factor for the group of patients treated with conventional chemotherapy (for BCSS: HR = 0.67, 95% CI = 0.50 to 0.89, *P* = 0.006; for RFS: HR = 0.69, 95% CI = 0.53-0.90, *P* = 0.005), but treatment was not randomly assigned in our study cohort. Formal prospective-retrospective studies of randomized trials [49], using locked-down assay and interpretation methodology based on cohort studies such as ours, would be the next step to determine, with a higher level of evidence, the relationship between chemotherapy, immune infiltrates and clinical outcome of cancer patients.

## Conclusions

The results from this large study indicate that the prognosis of FOXP3+ regulatory lymphocyte infiltration into breast tumors differs with the expression status of ER, HER2 and concurrent CD8+ T-cell infiltration. FOXP3+ TILs are significantly associated with poor survival in ER + breast cancers lacking cytotoxic T-cell infiltrates. In contrast, tumor regulatory T-cell infiltration is an independent and favorable indicator of survival in breast cancer patients who have HER2+/ER– breast tumors with CD8+ T-cell infiltrates.

## Authors’ contributions

SLi managed the study project, analyzed data and drafted the manuscript. WDF helped with study design, data analysis, and edited the manuscript. SLe significantly contributed to data management, statistical analysis and interpretation of the data. DG and SLa significantly contributed to acquisition and interpretation of the data. ZK contributed to acquisition and interpretation of the data, and edited the manuscript. TON led data generation and analysis, and edited the manuscript. WDF and TON organized the study. All authors read and approved the final version of the manuscript, and take responsibility for the work.

## Additional files

## Electronic supplementary material

Additional file 1: **Supplemental tables.** **Table S1.** Shows the selected cutoffs of TILs from the training set and the mean of the Youden Index (95% CI) obtained from the test set for each cutoff in the 100-time repeated runs. **Table S2.** Demonstrates the detailed results of FOXP3+ iTIL for BCSS from univariate and multivariate Cox regression analysis for the whole study cohort. **Tables S3-S5.** Shows the hazard ratios of FOXP3+ iTIL for RFS in the whole study cohort, ER+, HER2+/ER– and core basal subtypes. **Table S6.** Shows the distribution and FOXP3+ sTIL and tTIL in relation to patient clincopathologic characteristics. **Table S7.** Shows the hazard ratios of FOXP3+ iTIL for BCSS and RFS in multivariate analysis for patients treated with chemotherapy. (DOC 212 KB)

Additional file 2: **FOXP3+ TILs in breast cancer.** **Figure S1.** Shows some examples of FOXP3+ iTILs and sTILs in a breast cancer tissue microarray core (scale bar: 50 μm). (PDF 2 MB)

Below are the links to the authors’ original submitted files for images.

Authors’ original file for figure 1

Authors’ original file for figure 2

Authors’ original file for figure 3

## Abbreviations

5NP: five-marker negative phenotype
AJCC: American Joint Committee on Cancer
AST: adjuvant systemic therapy
BCSS: breast cancer-specific survival
CI: confidence interval
CK: cytokeratin
EGFR: epidermal growth factor receptor
ER: estrogen receptor
HER2: human epidermal growth factor receptor-2
HR: hazard ratio
IHC: immunohistochemical
iTIL: intratumoral tumor-infiltrating lymphocytes
PR: progesterone receptor
RFS: relapse-free survival
ROC: receiver operating characteristic
sTIL: stromal tumor infiltrating lymphocyte
TILs: tumor-infiltrating lymphocytes
TMA: tissue microarrays
TNP: triple-negative phenotype
Treg: regulatory T cells
tTIL: total tumor-infiltrating lymphocyte
