# Supplementary material for: Prognostic significance of FOXP3+ tumor-infiltrating lymphocytes in breast cancer depends on estrogen receptor and human epidermal growth factor receptor-2 expression status and concurrent cytotoxic T-cell infiltration
Source: Breast Cancer Res. 2014 Sep 6;16:432. doi: 10.1186/s13058-014-0432-8 (PMC4303113; doi:10.1186/s13058-014-0432-8)
Supplement: Supplementary file 1 — Additional file 1: Supplemental tables. Table S1. Shows the selected cutoffs of TILs from the training set and the mean of the Youden Index (95% CI) obtained from the test set for each cutoff in the 100-time repeated runs. Table S2. Demonstrates the detailed results of FOXP3+ iTIL for BCSS from univariate and multivariate Cox regression analysis for the whole study cohort. Tables S3-S5. Shows the hazard ratios of FOXP3+ iTIL for RFS in the whole study cohort, ER+, HER2+/ER– and core basal subtypes. Table S6. Shows the distribution and FOXP3+ sTIL and tTIL in relation to patient clincopathologic characteristics. Table S7. Shows the hazard ratios of FOXP3+ iTIL for BCSS and RFS in multivariate analysis for patients treated with chemotherapy. (DOC 212 KB) [file 13058_2014_432_MOESM1_ESM.doc]

**Supplemental tables:**

**Table S1. Cut-offs of iTIL, sTIL and tTIL from the training set, and the corresponding Youden Index in the** test set

| **Cut-off from training set** | **iTIL** | | **sTIL** | | **tTIL** | |
| --- | --- | --- | --- | --- | --- | --- |
|  | **number of runs** | **Mean of Youden Index from test set (95% CI)** | **number of runs** | **Mean of Youden Index from test set (95% CI)** | **number of runs** | **Mean of Youden Index from test set (95% CI)** |
| ≥1 | 24 | -0.007  (-0.068 – 0.024) | 1 | -0.035 | 1 | -0.033 |
| ≥2 | **42** | **0.008**  **(-0.037 – 0.041)** | 17 | 0.017  (-0.018 – 0.045) | 0 | NA |
| ≥3 | 3 | -0.022  (-0.031 – -0.011) | **26** | **0.032**  **(-0.029 – 0.081)** | 18 | 0.036  (-0.029 – 0.091) |
| ≥4 | 1 | -0.063 | 16 | 0.019  (-0.033 – 0.071) | **54** | **0.050**  **(0.005 – 0.098)** |
| ≥5 | 0 | NA | 16 | 0.014  (-0.038 – 0.050) | 11 | 0.032  (-0.014 – 0.070) |
| ≥6 | 0 | NA | 2 | -0.014  (-0.036 – 0.008) | 4 | 0.042  (0.012 – 0.064) |
| ≥7 | 1 | -0.003 | 0 | NA | 2 | -0.027  (-0.043 – -0.011) |
| ≥8 | 14 | 0.002  (-0.016 – 0.017) | 1 | -0.026 | 0 | NA |
| ≥9 | 0 | NA | 5 | -0.039  (-0.059 – -0.036) | 0 | NA |
| ≥10 | 0 | NA | 2 | -0.013  (-0.029 – 0.002) | 2 | -0.035  (-0.050 – -0.020) |
| ≥11 | 8 | 0.003  (-0.003 – 0.011) | 2 | -0.003  (-0.010 – 0.004) | 0 | NA |
| ≥12 | 3 | -0.006  (-0.012 – -0.002) | 0 | NA | 2 | -0.063  (-0.084 – -0.043) |
| ≥15 | 0 | NA | 1 | -0.036 | 1 | -0.026 |
| ≥18 | 0 | NA | 0 | NA | 2 | -0.006  (-0.010 – -0.002) |
| ≥19 | 1 | 0.002 | 0 | NA | 0 | NA |
| ≥20 | 1 | 0.006 | 0 | NA | 0 | NA |
| ≥22 | 0 | NA | 1 | -0.009 | 0 | NA |
| ≥24 | 1 | -0.003 | 0 | NA | 0 | NA |
| ≥25 | 0 | NA | 2 | -0.018  (-0.025 – -0.010) | 0 | NA |
| ≥29 | 0 | NA | 1 | -0.020 | 0 | NA |
| ≥32 | 0 | NA | 3 | -0.010  (-0.017 – 0.002) | 0 | NA |
| ≥33 | 0 | NA | 2 | 0.007  (0.005 – 0.009) | 0 | NA |
| ≥42 | 0 | NA | 1 | -0.015 | 1 | -0.013 |
| ≥100 | 0 | NA | 0 | NA | 1 | -0.005 |

NA: the optimal cutoff point was not available from that training-test run.

**Table S2.**

**Hazard ratio of FOX3+ iTIL for BCSS with univariate and multivariate analysis in the whole cohort**

| **Variable** | **Univariate analysis** | | **Multivariate analysis**  **n=3031** | |
| --- | --- | --- | --- | --- |
|  | **HR (95% CI)** | **p** | **HR (95% CI)** | **p** |
| **Age**  ≥ 50 vs. < 50 | 0.85 (0.75 - 0.96) | 0.011 | 1.06 (0.92 - 1.22) | 0.441 |
| **Grade**  3 vs. (1 and 2) | 2.12 (1.87 - 2.41) | < 0.001 | 1.59 (1.37 - 1.87) | < 0.001 |
| **Tumor size (cm)**  > 2 vs. ≤ 2 cm | 2.17 (1.92 - 2.45) | < 0.001 | 1.63 (1.42 - 1.88) | < 0.001 |
| **Nodal Status**  Positive vs. negative | 2.79 (2.48 - 3.15) | < 0.001 | 2.08 (1.79 - 2.43) | < 0.001 |
| **LVI**  Positive vs. negative | 2.25 (1.99 - 2.54 ) | < 0.001 | 1.33 (1.13 - 1.55) | < 0.001 |
| **Subtype** |  |  |  | |
| Luminal B vs. luminal A | 2.08 (1.78 - 2.45) | < 0.001 | 1.66 (1.38 - 2.00) | < 0.001 |
| HER2+/ER– vs. luminal A | 2.98 (2.40 - 3.70) | < 0.001 | 2.42 (1.89 - 3.08) | < 0.001 |
| Core basal vs. luminal A | 2.30 (1.87 - 2.84) | < 0.001 | 1.97 (1.54 - 2.51) | < 0.001 |
| 5NP vs. luminal A | 1.65 (1.30 - 2.10) | 0.002 | 1.75 (1.15 - 2.65) | 0.008 |
| **FOXP3+ iTIL**  ≥ 2 vs. <2 | 1.13 (0.99 - 1.29) | 0.073 | 0.89 (0.77 - 1.02) | 0.102 |

**Table S3.**

**Hazard ratio of FOX3+ iTIL for RFS with univariate and multivariate analysis** in the whole cohort

| **Variable** | **Univariate analysis**  **HR (95% CI)** | **Multivariate analysis**  **HR (95% CI) (n=3032)** |
| --- | --- | --- |
| Age (≥ 50 vs. < 50) | 0.80 (0.72 - 0.89) | 0.82 (0.81 - 1.05) |
| Grade (3 vs. 1 + 2) | 1.79 (1.60 - 1.99) | 1.42 (1.25 - 1.62) |
| Tumor size ( > 2 cm vs. ≤ 2 cm) | 1.81 (1.63 - 2.00) | 1.39 (1.23 - 1.57) |
| Nodal Status (Positive vs. negative) | 2.22 (2.00 - 2.47) | 1.76 (1.54 - 2.01) |
| LVI (Positive vs. negative) | 1.86 (1.68 - 2.07 ) | 1.20 (1.05 - 1.37) |
| Subtype |  |  |
| Luminal B vs. luminal A | 1.70 (1.48 - 1.96) | 1.44 (1.24 - 1.69) |
| HER2/ER– vs. luminal A | 2.35 (1.93 - 2.86) | 2.05 (1.65 - 2.54) |
| Core basal vs. luminal A | 1.79 (1.48 - 2.16) | 1.56 (1.26 - 1.94) |
| 5NP vs. luminal A | 1.25 (1.01 - 1.55) | 1.27 (0.86 - 1.87) |
| Foxp3+ iTIL (≥ 2 vs. < 2) | 1.06 (0.94 - 1.19) | 0.88 (0.77 - 1.00) |

**Table S4.**

Hazard ratio for RFS with multivariate analysis in ER+ group, and that with CD8+ iTIL status

| **Variable** | **ER+**  **(n = 2169)** | | **With CD8+ iTIL = 0**  **(n = 1477)** | | **With CD8+ iTIL ≥ 1**  **(n = 627 )** | |
| --- | --- | --- | --- | --- | --- | --- |
|  | **HR (95% CI) p** | | **HR (95% CI) p** | | **HR (95% CI) p** | |
| **Age** | 0.84 | 0.025 | 0.79 | 0.016 | 0.904 | 0.482 |
| ≥ 50 vs. < 50 | (0.72 - 0.98) |  | (0.66 - 0.96) |  | (0.68 - 1.20) |  |
| **Grade** | 1.46 | <0.001 | 1.41 | <0.001 | 1.57 | 0.001 |
| 3 vs. (1 and 2) | (1.37 - 1.69) |  | (1.19 - 1.68) |  | (1.19 - 2.07) |  |
| **Tumor size** | 1.41 | <0.001 | 1.34 | 0.001 | 1.57 | 0.001 |
| > 2 cm vs. ≤ 2 cm | (1.22 - 1.63) |  | (1.12 - 1.60) |  | (1.20 - 2.06) |  |
| **Nodal Status** | 1.66 | <0.001 | 1.80 | <0.001 | 1.35 | 0.044 |
| Positive vs. negative | (1.42 - 1.95) |  | (1.48 - 2.19) |  | (1.01 - 1.81) |  |
| **LVI** | 1.16 | 0.069 | 1.17 | 0.114 | 1.12 | 0.447 |
| Positive vs. negative | (0.99 - 1.37) |  | (0.96 - 1.43) |  | (0.84 - 1.50) |  |
| **Foxp3+ iTIL** | 1.02 | 0.856 | 1.15 | 0.103 | 0.90 | 0.407 |
| ≥ 2 vs. < 2 | (0.87 - 1.19) |  | (0.93 - 1.42) |  | (0.69 - 1.16) |  |

**Table S5. Hazard ratio for RFS with multivariate analysis in HER2+/ER– and core basal subgroups,**

and that with CD8+ iTIL status

| **Variable** | **Whole subgroup** | | **With CD8+ iTIL = 0** | | **With CD8+ iTIL ≥ 1** | |
| --- | --- | --- | --- | --- | --- | --- |
|  | **HR (95% CI) p** | | **HR (95% CI) p** | | **HR (95% CI) p** | |
| **HER2+/ER–** |  | | | | | |
| Age | 1.10 | 0.621 | 1.18 | 0.502 | 0.67 | 0.268 |
| ≥ 50 vs. < 50 | (0.75 - 1.64) |  | (0.92 - 1.94) |  | (0.32 - 1.37) |  |
| Grade | 1.87 | 0.018 | 1.78 | 0.070 | 1.69 | 0.290 |
| 3 vs. (1 and 2) | (1.11 - 3.13) |  | (0.96 - 3.32) |  | (0.64 - 4.44) |  |
| Tumor size | 1.47 | 0.059 | 1.21 | 0.442 | 2.36 | 0.017 |
| > 2 cm vs. ≤ 2 cm | (0.98 - 2.20) |  | (0.74 - 1.99) |  | (1.17 - 4.76) |  |
| Nodal Status | 1.73 | 0.018 | 0.93 | 0.824 | 4.26 | <0.001 |
| Positive vs. negative | (1.10 - 2.72) |  | (0.50 - 1.73) |  | (1.99 - 9.11) |  |
| LVI | 1.22 | 0.379 | 1.54 | 0.168 | 1.19 | 0.624 |
| Positive vs. negative | (0.78 - 1.92) |  | (0.83 - 2.82) |  | (0.60 - 2.36) |  |
| Foxp3+ iTIL | 0.78 | 0.205 | 0.96 | 0.862 | 0.46 | 0.028 |
| ≥ 2 vs. < 2 | (0.54 - 1.14) |  | (0.57 - 1.59) |  | (0.23 - 0.92) |  |
| **Core basal** |  | | | | | |
| Age | 1.05 | 0.789 | 0.78 | 0.308 | 1.63 | 0.122 |
| ≥ 50 vs. < 50 | (0.73 - 1.51) |  | (0.49 - 1.26) |  | (0.88 - 3.02) |  |
| Grade | 1.48 | 0.202 | 1.65 | 0.165 | 2.34 | 0.247 |
| 3 vs. (1 and 2) | (0.81 - 2.70) |  | (0.81 - 3.36) |  | (0.55 - 9.86) |  |
| Tumor size | 1.44 | 0.050 | 2.16 | 0.002 | 0.92 | 0.794 |
| > 2 cm vs. ≤ 2 cm | (1.00 - 2.48) |  | (1.33 - 3.50) |  | (0.50 - 1.71) |  |
| Nodal Status | 1.89 | 0.002 | 2.56 | <0.001 | 1.34 | 0.384 |
| Positive vs. negative | (1.27 - 2.82) |  | (1.51 - 4.35) |  | (0.69 - 2.58) |  |
| LVI | 1.27 | 0.244 | 0.79 | 0.379 | 2.75 | 0.003 |
| Positive vs. negative | (0.85 - 1.89) |  | (0.47 - 1.33) |  | (1.40 - 5.40) |  |
| Foxp3+ iTIL | 0.66 | 0.025 | 0.82 | 0.415 | 0.96 | 0.893 |
| ≥ 2 vs. < 2 | (0.46 - 0.95) |  | (0.51 - 1.32) |  | (0.49 - 1.85) |  |

**Table S6**. Clinico-pathologic characteristics and distribution of sTIL and tTIL in the study population

| **Characteristics** | **No. patients (%)** | **sTILs (≥ 3)** | | **tTILs (≥ 4)** | |
| --- | --- | --- | --- | --- | --- |
|  |  | **%** | **p-value** | **%** | **p-value** |
| **Age** |  |  | <0.001 |  | <0.001 |
| < 40 | 294 (7.4) | 64.3 (164/255) |  | 64.7 (165/255) |  |
| 40-49 | 844 (20.1)) | 63.9 (438/685) |  | 62.5 (428/685) |  |
| 50-65 | 1,425 (35.7) | 54.7 (649/1186) |  | 52.5 (623/1186) |  |
| > 65 | 1,429 (35.8) | 49.9 (574/1151) |  | 49.5 (570/1151) |  |
| **Grade** |  |  | <0.001 |  | <0.001 |
| 1 | 209 (5.2) | 38.6 (59/153) |  | 48.6 (56/153) |  |
| 2 | 1,563 (39.2) | 49.1 (633/1288) |  | 47.0 (606/1288) |  |
| 3 | 2,040 (51.1) | 62.7 (1065/1699) |  | 62.0 (1053/1699) |  |
| unknown | 180 (4.5) |  |  |  |  |
| **Tumor size (cm)** |  |  | 0.467 |  | 0.235 |
| ≤ 2 | 2,078 (52.1) | 55.2 (937/1697) |  | 53.8 (913/1697) |  |
| > 2-5 | 1,667 (41.8) | 56.7 (791/1394) |  | 56.0 (780/1394) |  |
| > 5 | 221 (5.5) | 52.4 (88/168) |  | 50.0 (84/168) |  |
| unknown | 26 (0.6) |  |  |  |  |
| **Nodal status** |  |  | 0.010 |  | 0.040 |
| Negative | 2,265 (56.7) | 53.8 (998/1856) |  | 53.0 (983/1856) |  |
| Positive | 1,719 (43.1) | 58.3 (824/1414) |  | 56.6 (800/1414) |  |
| unknown | 8 (0.2) |  |  |  |  |
| **LVI** |  |  | 0.003 |  | 0.009 |
| Negative | 2,106 (52.8) | 58.5 (833/1423) |  | 56.9 (809/1423) |  |
| Positive | 1,710 (42.8) | 53.3 (917/1720) |  | 52.2 (897/1720) |  |
| Unknown | 176 (4.4) |  |  |  |  |
| **AJCC stage** |  |  | 0.201 |  | 0.015 |
| I | 1,393 (34.9) | 53.7 (608/1132) |  | 52.8 (598/1132) |  |
| II | 2,255 (56.5) | 57.1 (1068/1872) |  | 55.6 (1041/1872) |  |
| III | 317 (7.9) | 55.3 (141/255) |  | 54.9 (140/255) |  |
| Unknown/missing | 27 (0.7) |  |  |  |  |
| **AST** |  |  | <0.001 |  | <0.001 |
| No AST | 1,676 (42.0) | 54.0 (749/1386) |  | 53.4 (740/1386) |  |
| Tamoxifen only | 1,276 (32.0) | 50.8 (527/1037) |  | 48.8 (506/1037) |  |
| Chemotherapy only | 727 (18.2) | 64.8 (391/603) |  | 62.7 (378/603) |  |
| Tamoxifen+Chemo | 297 (7.4) | 63.3 (150/237) |  | 64.6 (153/237) |  |
| Other | 16 (0.4) | 57.1 (8/14) |  | 64.3 (9/14) |  |
| **ER** |  |  | <0.001 |  | <0.001 |
| Negative | 1,200 (30.1) | 64.8 (589/909) |  | 64.1 (583/909) |  |
| Positive | 2,769 (69.1) | 52.3 (1232/2354) |  | 50.9 (1199/2354) |  |
| Unknown | 31 (0.8) |  |  |  |  |
| **HER2** |  |  | <0.001 |  | <0.001 |
| Negative | 3,316 (83.1) | 52.8 (1466/2779) |  | 51.6 (1434/2779) |  |
| Positive | 498 (12.5) | 76.7 (333/434) |  | 75.1 (326/434) |  |
| Unknown | 178 (4.4) |  |  |  |  |
| **Subtype** |  |  | <0.001 |  | <0.001 |
| Luminal A | 1,518 (38.0) | 45.6 (604/1325) |  | 43.5 (577/1325) |  |
| Luminal B | 829 (20.8) | 61.0 (457/749) |  | 61.7 (462/749) |  |
| Luminal/HER2 | 224 (5.6) | 77.0 (154/200) |  | 75.5 (151/200) |  |
| Luminal not assigned | 244 (6.1) | 39.6 (59/149) |  | 32.9 (49/149) |  |
| HER2+/ER– | 250 (6.3) | 77.5 (172/222) |  | 75.7 (168/222) |  |
| TNP | 630 (15.8) | 63.8 (335/525) |  | 64.0 (336/525) |  |
| Core basal | 330 (8.3) | 68.5 (207/302) |  | 68.9 (208/302) |  |
| 5NP | 162 (4.1) | 57.7 (79/137) |  | 58.4 (80/137) |  |
| Not assignable | 138 (3.4) | 57.0 (49/86) |  | 55.8 (48/86) |  |
| Other | 297 (7.4) | 41.1 (44/107) |  | 40.2 (43/107) |  |
| **CD8+ iTIL** |  |  | <0.001 |  | <0.001 |
| 0 | 3,316 (83.1) | 46.7 (978/2094) |  | 44.2 (925/2094) |  |
| ≥ 1 | 498 (12.5) | 75.3 (786/1044) |  | 76.6 (800/1044) |  |
| Unknown | 178 (4.4) |  |  |  |  |
| **Total** | **3,992 (100)** | **55.7 (1825/3277)** |  | **54.5 (1786/3277)** |  |

**Table S7. Hazard ratio for BCSS and RFS in the cases receiving adjuvant chemotherapy (multivariate analysis)**

| **Variable** | **For BCSS (n = 571)**  **HR (95% CI) p** | **For RSF (n = 572)**  **HR (95% CI) p** |
| --- | --- | --- |
| Age (≥50 vs. < 50) | 0.89 (0.63 - 1.26) 0.514 | 0.88 (0.65 - 1.21) 0.438 |
| Grade (3 vs. 1 + 2) | 1.80 (1.29 - 2.51) 0.001 | 1.36 (1.03 - 1.81) 0.031 |
| Tumor size ( > 2 cm vs. ≤ 2 cm) | 1.68 (1.26 - 2.25) 0.001 | 1.41 (1.09 - 1.82) 0.008 |
| Nodal Status (Positive vs. negative) | 2.06 (1.43 - 2.94) <0.001 | 1.98 (1.44 - 2.70) <0.001 |
| LVI (Positive vs. negative) | 1.61 (1.12 - 2.32) 0.010 | 1.27 (0.94 - 1.72) 0.119 |
| Subtype |  |  |
| Luminal B vs. luminal A | 1.68 (1.13 - 2.50) 0.010 | 1.48 (1.05 - 2.08) 0.024 |
| HER2/ER– vs. luminal A | 2.41 (1.53 - 3.78) <0.001 | 1.87 (1.25 - 2.80) 0.002 |
| Core basal vs. luminal A | 2.41 (1.54 - 3.78) <0.001 | 1.79 (1.20 - 2.65) 0.004 |
| 5NP vs. luminal A | 1.98 (0.92 - 4.26) 0.080 | 1.27 (0.60 - 2.69) 0.525 |
| Foxp3+ iTIL (≥ 2 vs. < 2) | 0.67 (0.50 - 0.89) 0.006 | 0.69 (0.53 - 0.90) 0.005 |
