# Supplementary material for: Prognostic significance of FOXP3+ tumor-infiltrating lymphocytes in breast cancer depends on estrogen receptor and human epidermal growth factor receptor-2 expression status and concurrent cytotoxic T-cell infiltration
Source: Breast Cancer Res. 2014 Sep 6;16:432. doi: 10.1186/s13058-014-0432-8 (PMC4303113; doi:10.1186/s13058-014-0432-8)
Supplement: Supplementary file 2 — Additional file 2: FOXP3+ TILs in breast cancer. Figure S1. Shows some examples of FOXP3+ iTILs and sTILs in a breast cancer tissue microarray core (scale bar: 50 μm). (PDF 2 MB) [file 13058_2014_432_MOESM2_ESM.pdf]

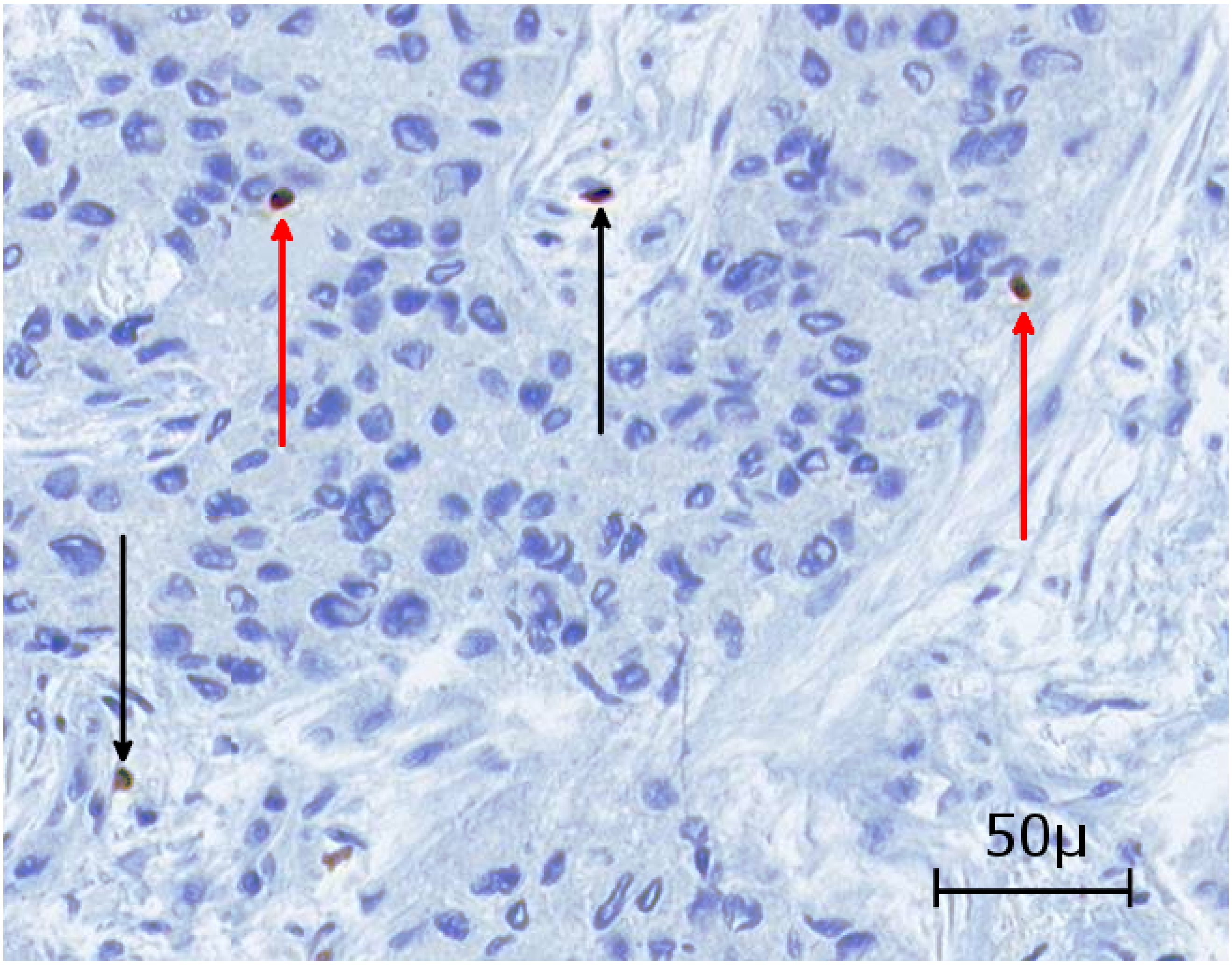

Figure S1. FOXP3+ TILs in breast cancer. Red arrows show examples of FOXP3+ iTIL, and black arrows indicate examples of FOXP3+ sTIL. All of the FOXP3-stained images are available at http://www.gpecimage.ubc.ca.
