# Supplementary figures and images for: Prognostic significance of FOXP3+ tumor-infiltrating lymphocytes in breast cancer depends on estrogen receptor and human epidermal growth factor receptor-2 expression status and concurrent cytotoxic T-cell infiltration
Source: Breast Cancer Res. 2014 Sep 6;16:432. doi: 10.1186/s13058-014-0432-8 (PMC4303113; doi:10.1186/s13058-014-0432-8)

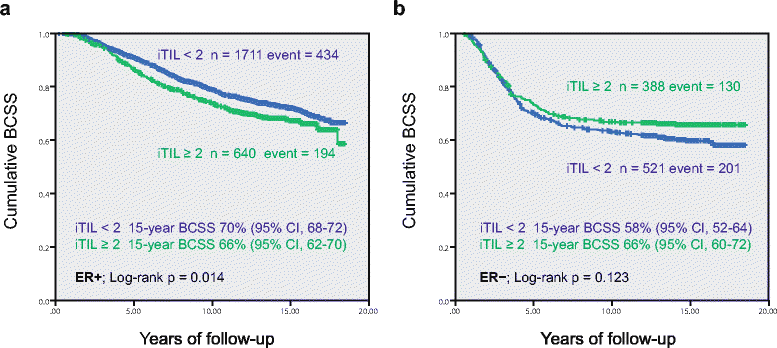

Supplement: Supplementary file 3 — Authors’ original file for figure 1 [file 13058_2014_432_MOESM3_ESM.gif]

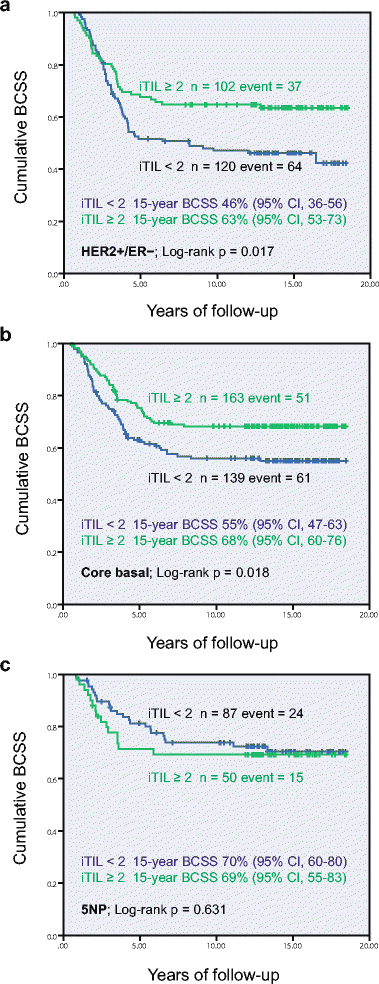

Supplement: Supplementary file 4 — Authors’ original file for figure 2 [file 13058_2014_432_MOESM4_ESM.gif]

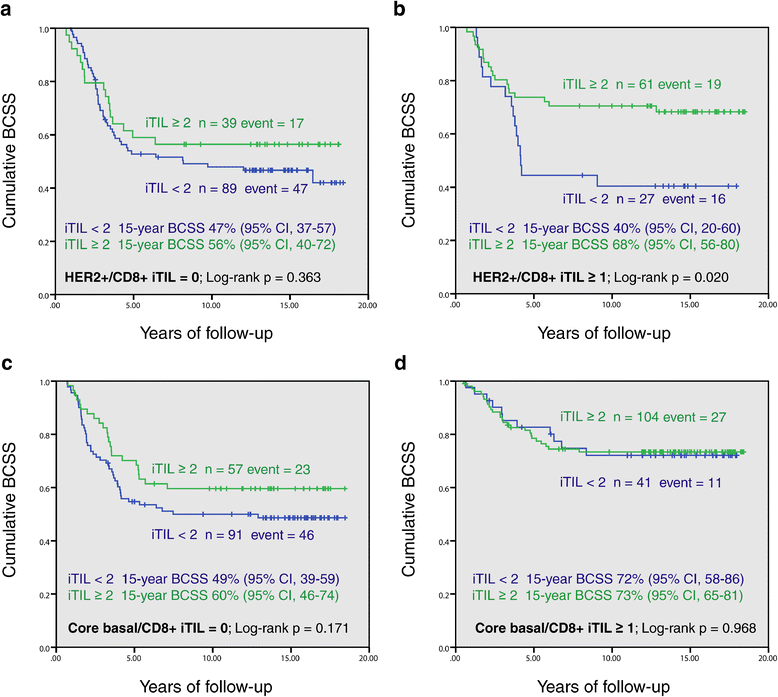

Supplement: Supplementary file 5 — Authors’ original file for figure 3 [file 13058_2014_432_MOESM5_ESM.gif]
